# Nucleic Acid–Based Therapies for Atherosclerosis

**DOI:** 10.1007/s11883-020-0826-2

**Published:** 2020-02-07

**Authors:** Petri Mäkinen, Anna-Kaisa Ruotsalainen, Seppo Ylä-Herttuala

**Affiliations:** 1 A.I. Virtanen Institute, University of Eastern Finland, Neulaniementie 2, P.O. Box 1627, 70211 Kuopio, Finland; 2 Heart Center and Gene Therapy Unit, Kuopio University Hospital, Kuopio, Finland

**Keywords:** Atherosclerosis, Nucleic acid, RNA, Antisense oligonucleotide, siRNA

## Abstract

**Purpose of Review:**

Atherosclerosis is characterized by accumulation of lipids and chronic inflammation in medium size to large arteries. Recently, RNA-based antisense oligonucleotides (ASOs) and small interfering RNAs (siRNAs) are being developed, along with small molecule-based drugs and monoclonal antibodies, for the treatment of risk factors associated with atherosclerosis. The purpose of this review is to describe nucleic acid–based therapeutics and introduce novel RNAs that might become future tools for treatment of atherosclerosis.

**Recent Findings:**

RNA-based inhibitors for PCSK9, Lp(a), ApoCIII, and ANGPTL3 have been successfully tested in phase II–III clinical trials. Moreover, multiple microRNA and long non-coding RNAs have been found to reduce atherogenesis in preclinical animal models.

**Summary:**

Clinical trials especially with ASOs and siRNAs directed to liver, targeting cholesterol and lipoprotein metabolism, have shown promising results. Additional research in larger patient cohorts is needed to fully evaluate the therapeutic potential of these new drugs.

## Introduction

Atherosclerosis is a multifactorial disease, triggered mainly by abundant accumulation of apolipoprotein B (ApoB)–containing lipoproteins and chronic vascular inflammation [1]. Atherosclerosis develops slowly over several decades, starting in young adults or even in early childhood [2]. Clinical complications result from advanced lesions, which are highly vulnerable and prone to rupture, intraplaque hemorrhages, and thrombus formation [3]. These most common complications of atherosclerosis account for ~ 70% of fatal acute myocardial infarctions, sudden coronary deaths, and strokes [4–7]. Despite of the development of potential new therapies and the improved treatment of high plasma lipid levels, cardiovascular diseases are still the leading cause of death worldwide, and the number of deaths is predicted to increase in the coming decades [4, 8]. Thus, there is a clear need for new treatment strategies and novel therapeutic agents, as the current treatments of atherosclerosis are mostly focused on the plasma lipid lowering. New approaches are focused at resolving the prevailing vascular inflammation and treating hypertension among other risk factors.

Lately, nucleic acid–based therapies have been developed and shown promising potential for the treatment of several diseases, even in the previously intractable ones. Several clinical trials have already proven efficacy of these therapeutics in the field of cardiovascular disease (Table 1). RNA-based therapeutics include small interfering RNAs (siRNAs), which are short double-stranded RNA molecules, that mediate mRNA degradation by binding to the complementary mRNA target sequence. Antisense oligonucleotides (ASOs) differ from siRNAs being single-stranded RNA or DNA molecules, but they also bind to the complementary target mRNA sequence and consequently prevent protein translation. Importantly, it has been noted that N-acetylgalactosamine (GalNAc) modification of ASOs increases the hepatic uptake significantly [9] and is therefore highly advantageous ASO/siRNA modification in cases where liver is the main target organ. MicroRNAs (miRNAs) are endogenous small non-coding RNA molecules, which bind to complementary mRNA or other targets in the genome. Function of miRNAs can be modulated, for example, by antagomirs, which are oligonucleotides preventing miRNA binding to its target site. Finally, long non-coding RNAs (lncRNAs) are endogenous over 200 nt RNA transcripts, that are not translated to proteins.

**Table 1:** Recent completed clinical trials with nucleic acid–based therapeutics

Drug name	Phase	Target molecule	Targeting approach	Main outcome	Trial no.	Reference
Mipomersen	III	ApoB	ASO	Up to 21% reduction LDL-C. Flu-like symptoms and hepatic transaminase increase as adverse effects.	NCT01475825	[11]
Inclisiran	II	PCSK9	GalNAc-siRNA	Up to 52.6% reduction in LDL-C. No serious adverse effects.	NCT02597127	[16••]
ANGPTL3-L_Rx_	I	ANGPTL3	GalNAc-ASO	Up to 63.1% reduction in TG. No serious adverse effects.	NCT02709850	[20•]
ISIS-APO(a)_Rx_	II	Lp(a)	ASO	Up to 71.6% reduction in Lp(a). Injection site effects as adverse effects.	NCT02160899	[25••]
IONIS-APO(a)-L_Rx_	I/IIa	Lp(a)	GalNAc-ASO	Up to 92% reduction in Lp(a). No serious adverse effects.	NCT02414594	[25••]
Volanesorsen	III	ApoC-III	ASO	Up to 77% TG reduction. Thrombocytopenia and injection site reactions as adverse effects.	NCT02211209, NCT02300233	[29••, 30]
AKCEA-APOCIII-L_Rx_	I/IIa	ApoC-III	GalNAc-ASO	Up to 77% TG reduction. No serious adverse effects.	NCT02900027	[31]

Nucleic acid therapeutics have been a promising novel tool in lipid lowering, through inhibition of function of a target gene, like proprotein convertase subtilisin kexin type 9 (PCSK9) [10]. However, multiple new potential targets for the regulation of plasma lipoprotein levels and vascular inflammation have been found. In addition, the discovery of new RNA classes has expanded the prospect of RNA molecules as novel therapeutic strategies. This review focuses on recent and novel nucleic acid–based therapies, which have advanced into clinical development during the past 3 years and describe also new promising therapeutic targets for atherogenesis.

## Liver-Directed Lipid-Lowering Therapies

As hyperlipidemia is a strong risk factor for atherosclerosis, several targets to control lipoprotein metabolism with nucleic acid directed therapeutics have been developed. To affect lipoprotein metabolism, one of the first and most obvious targets is apolipoprotein B (ApoB), the predominant apolipoprotein in LDL and VLDL particles. Mipomersen is ASO against ApoB. It has been approved by FDA for patients with familiar hypercholesterolemia (FH) since 2013, whereas the European Medicines Agency refused marketing authorization due to side effects, the most severe being liver damage (https://www.ema.europa.eu/en/documents/smop-initial/questions-answers-refusal-marketing-authorisation-kynamro-outcome-re-examination_en.pdf). Alternative dosing strategy was recently studied in FH patients, with the idea that injecting the compound thrice per week with lower amount instead of weekly injections might ease flu-like side effects while still maintaining the LDL-lowering effect [11]. However, injection site reactions were more common with this approach [11]. In addition, the MICA study reported a 22.6 ± 17.0% decrease in pre-apheresis LDL with mipomersen [12] in patients with maximal drug therapy and regular apheresis for hypercholesterolemia. Again, side effects were reported to be frequent [12]. Finally, mipomersen has been reported to decrease plasma Lp(a) in healthy subjects by 21% [13] and 27.7% in FH patients [11]. Previously, a clinical trial with ApoB targeting siRNA was terminated due to immune system activation [14]. These results raise safety concerns and suggest that RNA-based therapy against ApoB might not be a suitable approach for treatment of dyslipidemia.

PCSK9 plays an important role in LDL homeostasis by binding into LDL receptor promoting its degradation and preventing its recycling to the hepatocyte membrane. Thus, PCSK9 reduces the number of LDL receptors and increases LDL concentration in the plasma, making PCSK9 an attractive target for therapies lowering its expression. In addition to antibody and small molecule–based inhibition of PCSK9, multiple approaches focusing on small RNAs have been tested. Early trials with PCSK9 ASO were terminated due to renal tubular toxicity [15]. However, inclisiran, a GalNAc-conjugated siRNA against PSCK9, is studied in several phase III trials (NCT03705234, NCT03814187, NCT03400800, NCT03399370, NCT03397121, NCT03851705) for evaluating its effects on cardiovascular outcomes and FH. Previous phase II trial showed that inclisiran lowers PCSK9 and LDL levels in a dose-dependent manner in patients with elevated LDL cholesterol and at high cardiovascular risk [16••]. After 180 days, the mean LDL reductions ranged between 27.9 and 41.9% after a single dose, or 35.5–52.6% after two doses [16••]. Importantly, PCSK9 siRNA did not evoke any significant adverse effects and was well-tolerated during the study. Moreover, diabetes had no effect on the treatment effect [17]. It should be also noted that recent results published by a trial utilizing PCSK9 inhibition with monoclonal antibody alirocumab suggest that it might reduce death after acute coronary syndrome [18]. Also, a clinical trial with another monoclonal antibody against PCSK9, evolocumab, has shown similar results [19]. Therefore, PCSK9 is a highly attractive potential target, and future studies will further show the full potential of ASO-based PCSK9 inhibition in preventing cardiovascular diseases.

Angiopoietin-like protein 3 (ANGPTL3) acts as an inhibitor of lipoprotein and endothelial lipases, increasing triglyceride (TG), LDL, and HDL levels. It has been demonstrated that ASO against ANGPTL3 decreases TG levels up to 63% in humans [20•]. No serious adverse effects were reported with this GalNAc-conjugated ASO called IONIS-ANGPTL3-L_Rx_. A subsequent phase II study is underway in patients with hypertriglyceridemia, type 2 diabetes mellitus, and nonalcoholic fatty liver disease (NCT03371355). Recently, phase I studies with a monoclonal antibody against ANGPTL3 have shown to reduce TG levels up to 83% and to be well-tolerated [21], providing additional proof that ANGPTL3 blockade may serve as a novel treatment strategy especially in individuals with high TG levels. On the contrary, angiopoietin-like protein 4 (ANGPTL4) deficiency has been shown to increase atherogenesis via myeloid cell proliferation, foam cell formation, and vascular inflammation [22].

Lipoprotein (a) (Lp(a)) consists of apoliporotein (a) (Apo(a)) bound to ApoB of the LDL particle. Recent observations from epidemiological studies have shown that Lp(a) is an independent risk factor for cardiovascular diseases [23]. A phase I study with ISIS-APO(a)_Rx_, an ASO binding to apo(a) mRNA, reduced plasma levels by 39.6–77.8% in healthy volunteers [24]. A subsequent phase II trial showed similar 67–72% reductions in patients with elevated Lp(a) [25••]. At the same time, GalNAc-conjugated ASO with the same target sequence, IONIS-APO(a)-L_Rx_, was shown to reduce Lp(a) levels in humans up to 92% [25••] in healthy volunteers. Importantly, the effective dose to decrease Lp(a) by 50% was 30-fold less with the GalNAc-modified version of the ASO. A phase II study is currently ongoing to assess this ASO in patients with hyperlipoproteinemia (a) and established cardiovascular disease (NCT03070782) [26]. Since Lp(a) is not usually measured in standard clinical practice, more effective screening and larger clinical trials lowering Lp(a) are awaited to further evaluate Lp(a) as a therapeutic target.

Apoliporotein C-III (ApoC-III) is found in chylomicrons, VLDL, and remnant particles. It inhibits LPL activity. Plasma ApoC-III levels have been shown to independently predict cardiovascular mortality. A phase II study proved that reduction of apoC-III by ASO volanesorsen triggered a significant drop in plasma TGs in patients with hypertriglycemia [27]. Also, VLDL is lowered by volanesorsen [28]. Phase III studies with familial chylomicronemia syndrome and hypertriglycemia have been completed (NCT02211209, NCT02300233) [26] [29••]. TG levels were greatly reduced in both studies (77% and 73%), but injection site reactions were common side effects [29••, 30]. Volanesorsen received a negative decision by FDA in August 2018, but was followed by a positive opinion by EUs Committee for Medicinal Products for Human Use in March 2019. Consequently, volanesorsen has been approved in Europe for patients with genetically confirmed familial chylomicronemia syndrome (FCS) and at high risk for pancreatitis [30]. Furthermore, a GalNAc-conjugated ASO against ApoC-III, AKCEA-APO-CIII-L_Rx_, decreased TG levels in average up to 77% in phase I/IIa study in healthy volunteers. Importantly, this drug was reported to be well-tolerated [31]. A phase II study (NCT03385239) is currently active at recruiting stage with this ASO.

The current ASO and siRNA-based therapeutics have shown remarkably good and long-lasting results and are on their way to a portfolio of preventive treatments of lipid levels. However, it should be noted that the regulation of cholesterol homeostasis is more complex, and new potential targets for therapeutic interventions affecting cholesterol and lipoprotein metabolism are being discovered. For example, lncRNA called LASER has been implicated to participate in cholesterol homeostasis in hepatocytes [32]. Likely, there are other yet unidentified lncRNAs and other RNA species participating in this regulation as well.

## New Potential Targets in Atherogenesis

There are several critical steps in early atherogenesis, which might serve as targets for new therapies to prevent or regress vascular diseases. In the early stages of atherosclerosis, dysfunctional arterial endothelium allows excessive LDL to penetrate the endothelial layer and adhere to intimal proteoglycans via (ApoB100) and gradually accumulate into the intimal layer [33, 34]. Intimal LDL accumulation provokes the expression of adhesion molecules and chemotactic proteins from endothelial cells that enhances the adhesion of circulating monocytes and other inflammatory cells in the arterial wall. A few miRNAs, such as miR-126 and miR-155, have been shown to improve endothelial function and inhibit adhesion of leukocytes and thereby possess anti-atherogenic properties [35, 36]. Several other miRNAs are known to regulate proliferation and apoptosis of endothelial cells [37, 38], and affect endothelial cell senescence, which lead to endothelial dysfunction [39]. Interestingly, antagomirs directed against miR-92a prevents endothelial dysfunction and atherosclerotic lesion progression in mice [40]. Moreover, global run-on sequencing-based transcriptional profiling of hypoxic endothelial cells showed several non-coding RNAs which are differentially regulated in atherosclerotic plaques [41] and therefore, might be potential targets for RNA therapies. The inhibition of chemokine monocyte chemoattractant protein 1 (MCP-1) and its receptor chemokine (C-C Motif) receptor 2 (CCR2) have shown to inhibit plaque growth and monocyte accumulation in the plaque in RNAi-based gene silencing studies [42–45]. An interesting new inhibitor of MCP-1, emapticap pegol (NOX-E36), is a 40-nucleotide oligonucleotide aptamer that binds and inhibits MCP-1 with high affinity and specificity. It has been studied in a phase II trial in diabetic patients with albuminuria (NCT01547897) [46] and shown to drive macrophages towards an anti-inflammatory phenotype in mice [47]. A recent study showed that lncRNA MANTIS affects endothelial cell monocyte adhesion via ICAM-1 [48]. Furthermore, statins were shown to upregulate MANTIS. Thus, MANTIS might be involved in pleiotropic effects of statins [48]. MANTIS appears to be a multifunctional atheroprotective lncRNA awaiting further studies to explore its role in therapeutic applications.

In the intima, LDL undergoes oxidative modification and is taken up by macrophages that transform into foam cells, which accumulate in arterial intima forming fatty streaks [42, 49]. Cholesterol can be transported to and from macrophages by several mechanisms that are potential targets to inhibit atherosclerotic plaque formation and progression [50]. Macrophages express several pattern recognition receptors (PRRs), such as scavenger receptor A1 (SR-A1), macrophage receptor with collagenous structure (MARCO or SR-A2), CD36, scavenger receptor class B type 1 (SR-B1), lectin-like oxidized LDL receptor 1 (LOX-1), and CXCL16, which are scavenger receptors for oxidized LDL [51], but SR-A1 and CD36 mediate as much as 75–90% of modified LDL uptake in in vitro conditions. These receptors are important targets to inhibit macrophage foam cell formation, as well as plaque macrophage apoptosis, migration of monocytes, and plaque inflammatory responses [52]. It has been shown that macrophage scavenger receptor expression can be modulated with RNAi to exert an effect on development of atherosclerosis [53]. Moreover, macrophage reverse cholesterol transport is an interesting target, as several miRNAs have been found to regulate sterol-regulated transcription factors, liver X receptors α and β activated ATP-binding cassette (ABC) transporters ABCA1 and ABCG1 that mediate the transportation of free cholesterol to lipid-poor apolipoprotein A1 (apoAI) to form nascent or lapidated HDL particle [35, 54]. Lately, the role of lncRNAs in cholesterol homeostasis has been studied. Subsequently, a lncRNA named as LeXis regulates crosstalk between liver X receptor and SREBP and sustains hepatic sterol content and serum cholesterol levels [55•]. As a therapeutic approach, AAV-mediated overexpression of LeXis has been shown to lower serum cholesterol and TG levels, and furthermore reduce atherosclerosis burden [55•]. Additionally, the same group discovered another lncRNA MeXis, which regulates macrophage cholesterol efflux through ABCA1 [56]. Moreover, a primate-specific lncRNA CHROME elevated in atherosclerotic plaques has been identified, which is upregulated in response to increased cholesterol levels through LXR [57•]. Knockdown of CHROME reduces ABCA1 expression and increases several miRNAs involved in cholesterol metabolism. Thus, CHROME appears to play a key role in cholesterol homeostasis in humans among lncRNAs. Finally, a lncRNA AC096664.3 has been shown to play a role in PPARγ and ABCG1-mediated cholesterol signaling in smooth muscle cells [58], whereas lncRNA ENST00000602558.1 has been shown to regulate ABCG1 in vascular smooth muscle cells via nuclear factor κB (NF-κB) p65 [59]. This suggests that there are numerous RNAs involved in the cholesterol trafficking in macrophages and other cell types, which can serve as potential targets for future attempts to regulate cholesterol accumulation in order to affect atherogenesis.

Several previously discussed nucleic acid–based therapies targeted to ApoB, PCSK9, ANGPTL3, Lp(a), and ApoC-III are promising tools to resolve plaque inflammation via reduction in cholesterol levels. In hypercholesterolemic mouse models, reduction in plasma cholesterol reduces plaque inflammation [60–62]. In addition, recently, a high-fat diet-regulated miRNA gene network was found from baboons [63]. These miRNAs had discordant effects on plasma LDL concentration and might serve as potential targets for LDL lowering. Nevertheless, treatments targeted to plaque inflammation itself are still very limited. Local inflammatory stimulus within plaques activates macrophages, mast cells, and T cells to release cytokines that regulate cell migration and adhesion. In addition, pro-inflammatory stimulus in the vascular wall inhibits fibrotic cap formation by decreasing collagen synthesis and by increasing the amount of proteases that digest extracellular matrix proteins [42, 64]. Intracellular cholesterol and pro-inflammatory mediators induce the NF-κB pathway in plaque macrophages [65]. NF-κB is a master regulator of pro-inflammatory effects in the atheroma plaque, and thus, NF-κB-targeted therapies possess great potential to strike against atheroma plaque development and instability. Interestingly, miRNA-146a has shown great potential in inhibiting NF-κB activation and limiting the development of atherosclerotic plaques in mice [66]. MiRNA-210 is believed to enhance fibrous cap stability in atherosclerotic lesions [67]. A lncRNA MALAT1 has been linked to plaque inflammation, hematopoietic deficiency of MALAT1 promoting accumulation of inflammatory cells in lesions in an atherosclerotic mouse model [68•]. In humans, higher MALAT1 levels in plaques were shown to correlate with fewer forthcoming major adverse cardiovascular events [68•]. In addition, lncRNA called SENCR (smooth muscle and endothelial cell-enriched migration/differentiation-associated lncRNA) was found from human coronary artery smooth muscle cells by sequencing and shown to inhibit smooth muscle cell migration [69]. The long intergenic non-coding RNA-p21 (lincRNA-p21) is shown to regulate p53, a master regulator of cell proliferation and apoptosis, and thereby affecting atherogenesis. LincRNA-p21 is reduced in human coronary atherosclerosis and in a hypercholesterolemic mouse model [70]. In addition, its inhibition by lentivirus-driven supply of siRNA results in neointimal hyperplasia after carotid artery injury [70].

Genome-wide association studies have identified an antisense non-coding RNA ANRIL (antisense non-coding RNA in the INK4 locus), located at the chromosome 9 p21 locus, which is currently the most significant risk region for atherosclerosis and coronary artery disease. ANRIL has several effects in different vascular cell types, but underlying mechanisms how ANRIL affects atherogenesis are not known [71, 72]. In addition, multiple linear and circular splicing variants of ANRIL have shown to have pro- and anti-atherogenic effects, but the specific mechanisms need to be investigated [73]. ANRIL, like other ncRNAs, are able to regulate the expression of their neighboring genes via cis-trans gene regulation, and further influence microRNA signaling, thus providing multiple potential therapeutic targets for the treatment of atherosclerosis. Interestingly, the coronary artery disease risk associated with Chr9p21 and ANRIL seems to be independent of plasma cholesterol levels and other classical risk factors [74]. ANRIL has been proposed to regulate vascular cell inflammation, viability, proliferation, and senescence, thereby providing novelty for the future atherosclerosis therapies [71, 73, 75–77]. Overall, lncRNAs are promising targets for the development of new therapeutic approaches for cardiovascular diseases.

## CRISPR/Cas9 Gene Editing as a Novel Therapeutic Approach for Atherosclerosis?

Most of the abovementioned genes modulating lipid homeostasis and being targets for RNA therapeutics have been already explored with CRISPR/Cas9 gene editing technique. First, it was shown by 2 independent studies that CRISPR-mediated modification of PCSK9 led to over 50% or 80% editing after single administration [65, 66]. This modification reduced serum PSCK9 levels to undetectable levels and lowered cholesterol 30–40% in mice [78•, 79]. Recently, a knock-in mouse model with liver-specific expression of human PCSK9 has been developed [80]. In this model, CRISPR-mediated editing was used to individually affect human or mouse PCSK9 and alter cholesterol levels regulated by human PCSK9 [80]. Thus, this model might be suitable for studies where new therapies against PCSK9 are explored. LDL receptor CRISPR knockout led to severe hypercholesterolemia and atherosclerosis in mice [81]. Subsequently, when the same animals were knocked out for ApoB, the disease was completely eradicated. In addition, CRISPR/Cas9-mediated LDLR/ApoE-deficient pigs have been generated [82]. Moreover, CRISPR/Cas9 gene editing has been used to generate hyperlipidemic rabbits by knocking down LDL receptor alone or in combination with ApoE [83]. Moreover, ANGPTL3 has been interrupted with CRISPR/Cas9 system [84]. This led to lower ANGPTL3, TG, and cholesterol levels. It is clear that CRISPR/Cas9 gene editing will serve as a valuable research tool in atherosclerosis but might also offer new treatment possibilities via gene therapy approaches.

## Conclusions

In summary, multiple RNA-based and/or RNA-targeted therapeutics are now heading for phase III trials. Results of trials are eagerly waited. Moreover, CRIPSR/Cas9 gene modification technique has rapidly consolidated its role in creating models and therapies for cardiovascular diseases. This technique will provide generation of novel models useful in studying the pathogenesis and therapies of atherosclerosis. Also, when it comes to lncRNAs, the journey has just taken the first steps.
